# Predictive factors for Alzheimer’s disease progression: a comprehensive retrospective analysis of 3,553 cases with 211 months follow-up

**DOI:** 10.3389/fneur.2023.1239995

**Published:** 2023-08-24

**Authors:** Aynur Özge, Reza Ghouri, Nevra Öksüz, Bahar Taşdelen

**Affiliations:** ^1^Department of Neurology, School of Medicine, Mersin University, Mersin, Türkiye; ^2^Department of Biostatistics, School of Medicine, Mersin University, Mersin, Türkiye

**Keywords:** Alzheimer’s disease, dementia, neuroimaging, predictors, functional capacity, hippocampal atrophy, white matter intensities, Fazekas grading

## Abstract

**Background:**

There is conflicting data regarding the predictors of Alzheimer’s Disease (AD), the most common form of dementia. The main objective of the study is to evaluate potential predictors of AD progression using a comprehensive follow-up dataset that includes functional/cognitive assessments, clinical and neuropsychiatric evaluations, and neuroimaging biomarkers such as hippocampal atrophy or white matter intensities (WMIs).

**Method:**

A total of 161 AD cases were recruited from a dementia database consisting of individuals who consulted the Dementia Outpatient Clinic of the Neurology Department at Mersin University Medical Faculty between 2000 and 2022, under the supervision of the same senior author have at least 3 full evaluation follow-up visit including functional, clinical, biochemical, neuropsychological, and radiological screening. Data were exported and analyzed by experts accordingly.

**Results:**

Mean follow-up duration of study sample was 71.66 ± 41.98, min 15 to max 211 months. The results showed a fast and slow progressive subgroup of our AD cases with a high sensitivity (Entropy = 0.836), with a close relationship with several cofactors and the level of disability upon admittance. Hippocampal atrophy and WMIs grading via Fazekas were found to be underestimated predictors of AD progression, and functional capacity upon admittance was also among the main stakeholders.

**Conclusion:**

The study highlights the importance of evaluating multiple potential predictors for AD progression, including functional capacity upon admittance, hippocampal atrophy, and WMIs grading via Fazekas. Our findings provide insight into the complexity of AD progression and may contribute to the development of effective strategies for managing and treating AD.

## 1. Introduction

Dementia is a progressive decline in cognitive function caused by brain damage or disease, and it is one of the most significant causes of disability in the elderly worldwide. Alzheimer’s disease (AD) is a prevalent form of dementia, characterized by a gradual decline in cognitive abilities, including memory, language, and problem-solving skills. AD affects millions of individuals globally and is associated with a considerable socio-economic burden (1). The progression of dementia can vary depending on the type of dementia. For instance, AD typically advances slowly over several years, with symptoms worsening over time. Various factors have been suggested to accelerate the progression of AD, including age, genetics, vascular factors, lifestyle choices, and psychiatric comorbidities such as depression (2, 3). Additionally, medical disorders like hypertension, diabetes, and depression have been linked to faster dementia progression (4, 5), while social support may play a protective role in slowing the disease’s progression (6).

Neuropsychiatric evaluations are essential for assessing cognitive and behavioral symptoms in dementia patients. These evaluations involve various parameters, such as cognitive function measures, memory tests, executive function tests, language tests, visuospatial function tests, and behavioral symptoms scales (7, 8).

Biomarkers are also used to support the diagnosis of dementia, but they should be used in conjunction with other diagnostic tools and clinical evaluations as they do not provide a definitive diagnosis (9). Neuroimaging biomarkers, such as brain volume reduction in specific regions affected by dementia, amyloid deposition, and tau accumulation detected by PET or CSF biomarkers, and vascular changes supported by MRI perfusion imaging, provide valuable information about the progression of neurodegeneration (9, 10). Functional imaging techniques, like PET or fMRI, offer insights into the neural mechanisms underlying dementia symptoms (11, 12). However, it is essential to use neuroimaging biomarkers together with other diagnostic tools, such as cognitive assessments, clinical evaluations, and other biomarkers (13).

The significance of white matter ischemic lesions in dementia cases remains a topic of conflicting data. The Fazekas scale, commonly used to assess white matter hyperintensities (WMHs) on brain MRI scans, provides a rating system ranging from 0 to 3, with 0 indicating no WMHs and 3 indicating severe WMHs (14). Studies have indicated that individuals with higher Fazekas scores (more severe WMHs) are more likely to experience cognitive decline and dementia (15, 16). However, comprehensive, prospective data on the primary role of Fazekas grading in detecting dementia progression and its correlation with other biomarkers are lacking (17).

In clinical practice, not all individuals with AD progress to severe stages of the disease, and predicting who will progress rapidly remains challenging. Regular monitoring and management of risk factors can help slow the progression of early AD to late phases of dementia and improve the quality of life for patients and their caregivers (18, 19). However, identifying cases prone to rapid progression and providing appropriate support mechanisms remain crucial for clinicians.

The main objectives of this study were:

- To comprehensively evaluate the long-term progression of AD using a comprehensive dataset supervised by the same senior author.
- To differentiate cases of fast and slow progression of AD within the dataset.
- To identify potential cofactors that may influence disease progression and provide insights for further research.
- To identify the specific roles of hippocampal atrophy and white matter hyperintensities (WMIs) in the disease process.
- To discuss the most predictive batteries for assessing these issues.

## 2. Materials and methods

### 2.1. Data collection and patient selection

Patients for this study were recruited from a dementia database consisting of individuals who had consulted the Dementia Outpatient Clinic of the Neurology Department at Mersin University Medical Faculty between 2000 and 2022, under the supervision of the same senior author (AO). The data set included individuals from the following groups:

Healthy controls: individuals who were invited to participate in the study through our network or volunteers as any reason.

Prodromal dementia: relatives of known dementia cases who requested a check-up.

Preclinical dementia or mild cognitive impairment (MCI) cases: patients who presented with memory or other unimodal cognitive dysfunction and had functional impairment.

Clinical dementia cases: patients who were submitted or referred with relevant cognitive dysfunctions, including memory impairment.

This study included clinically diagnosed (excluding mixed dementias), legally consented participants with a definite diagnosis of Alzheimer’s disease, who underwent comprehensive neurological examination and neuropsychological test battery at least three times. Additionally, the participants had accessible MRI data and complete medical documentation available. Only clinically diagnosed Alzheimer’s patients with a Global Deterioration Scale (GDS) score greater than Grade 3 were included in the study. Diagnosis of AD was made according to the revised National Institute on Aging and Alzheimer’s Association (NIA-AA) criteria (9). Prodromal and preclinical cases were out of scope of the paper. After written approval of the ethics committee and of the institutions permission in which the study was to be performed were obtained the study started (decision no: 2023/28, date: 10.03.2023).

All patients were evaluated by the same clinic under the supervision of the same author (AO) with regular quarterly visits. Neuropsychiatric evaluation was generally performed every other visit (an average of 2 times per year) and recorded in the database. Additionally, each patient had several other visits under the scope of this study, concerning medical or medication-related symptoms. During each visit, the physician provided medication and laboratory tests, if necessary and performed. All patients were subjected to differential diagnosis and underwent a neurological examination followed by necessary laboratory procedures. Then, they received neuropsychological examination using the same methodology. The electronic data recording system in the electronic database of the Turkish Alzheimer’s Working Group^1^, which was developed under the leadership, was used for the following **neuropsychological evaluation** items:

1. **Functional capacity**: The Barthel Daily Living Activities Scale (BDLAS) and Elderly Daily Living Activity Scale (EDLAS) were used (20).
2. **Cognition:** Turkish validated Mini-Mental State Exam (MMSE) was used for a screening test (21).
3. **Numerical range:** The forward and backward digit-span test was used in our dataset. We used the following numerical sequences in our study: 28/51–372/494–5,169/6294–83,529/61074–285,164/917203–4,072,916/3508172 (22).
4. **Calculation:** In our study, we applied the following calculation problems: 5 + 3; 15 + 7; 31–8; 5 × 13; and 39/3. The maximum score for the correct completion of all arithmetic tasks was 5 (23).
5. **Abstraction:** The participants were asked to interpret three different proverbs. Each correct answer was worth 1 point. The proverbs used in this battery were “to be worn to the bone” (Tur. lit. “Getting black water on my feet”), “He that lies down with dogs will rise up with fleas” (Tur. lit. “Grapes grow darker by facing each other”) and “As the twig is bent so is the tree inclined” (Tur. lit. “The tree is only bent when ripe”). These were chosen among the most used Turkish proverbs (24).
6. **The Word Memory Test (WMT)**: This test series assess verbal learning and memory through three learning experiments, delayed recall, and recognition subtests. In the learning experiments, the examiner verbally presents a set of 10 neutral nouns in different sequences, such as oil, building, arm, beach, letter, cat, stick, ticket, grass, and motor, with no adjectives to avoid bias. Patients are then asked to recall all the words they remember from the set, and one point is awarded for each correctly counted word. In the delayed recall stage, conducted after three additional tests, patients are asked to recall the words they previously learned. In the next step, a mixed list of 20 words, including 10 new words of similar nature such as mosque, five, mountain, string, coffee, lira, slippers, soldier, hotel, and village, is presented to the patient, who is then asked to recognize the previously learned words. The total score for correct positive and false negative conditions in this stage is 20. During the administration of this test, a second pause is given before proceeding to the next task, and feedback about patient responses is not provided (25).
7. **Boston Naming Test (BNT):** It is a widely used neuropsychological test that assesses an individual’s ability to name objects. The BNT consists of 60-line drawings of objects of increasing difficulty, and the participant is asked to name each object. One point is awarded for each correctly named object (26).
8. **Clock Drawing Test (CDT)**: It is a widely used neuropsychological test that assesses an individual’s ability to draw a clock face and set the hands to a specified time. The CDT is scored on a 10-point scale based on the accuracy of the drawing and the placement of the hands (27).
9. **Grading of the disability**: The Global Deterioration Scale (**GDS**) for Alzheimer’s Disease is a widely used tool for staging the progression of cognitive decline in individuals with Alzheimer’s disease. The GDS is based on a seven-point scale that ranges from no cognitive impairment to severe cognitive decline as follows:

- Grade 1: No cognitive impairment.
- Grade 2: Questionable cognitive impairment.
- Grade 3: Mild cognitive impairment.
- Grade 4: Moderate cognitive impairment.
- Grade 5: Moderately severe cognitive impairment.
- Grade 6: Severe cognitive impairment.
- Grade 7: Very severe cognitive impairment.

The GDS is a widely used tool in clinical practice and research to measure the progression of Alzheimer’s disease. It provides a standardized way to assess the severity of cognitive decline and track changes over time (28).

The neuropsychological battery used in our study underwent meticulous adaptation and validation. Employing a standardized electronic data recording system, we utilized established assessment tools to ensure precision and reliability during the evaluation process. The battery covered diverse cognitive domains, such as functional capacity, cognition, numerical range, calculation, abstraction, verbal learning, memory, object naming, and clock drawing abilities. Additionally, we integrated the widely used GDS for Alzheimer’s Disease to grade the severity of cognitive decline in participants. Overall, this rigorous adaptation and validation process provided a robust foundation, ensuring the accuracy and validity of our neuropsychological data. Consequently, our study can yield valuable insights into cognitive impairments among different populations.

To distinguish AD from other causes of dementia, a neuroimaging protocol with MRI or CT was used for differential diagnosis. Some cases underwent standardized SPECT/PET investigations if necessary. High-resolution MRI scans were acquired using a 1.5 Tesla or higher field strength scanner. The imaging protocol included T1-weighted, T2-weighted, and fluid-attenuated inversion recovery (FLAIR) sequences. Hippocampal atrophy was evaluated using T1-weighted images, employing region-of-interest (ROI) analysis and manual tracing to measure hippocampal volumes. The measurements were normalized to intracranial volume or an appropriate reference structure. Fazekas grading for white matter lesions was performed using T2-weighted and FLAIR images, following the Fazekas scale to assign scores based on the distribution and intensity of lesions by the same author (AO).

This study included clinical dementia cases (GDS 3 or more) with at least three comprehensive evaluation visits, including eligible MRI scans for radiological evaluation, and whole biochemical screening to double-check.

Exclusion criteria included the presence of known inflammatory, infectious, or immune diseases that may cause cognitive disturbances, overlapping syndromes (AD plus vascular dementia, motor neuron disorders, etc.), comorbid neuropsychiatric disorders (e.g., epilepsy, previously known psychotic disorders, dependency, etc.), major head trauma, severe renal or hepatic failure, recent severe hemodynamic disturbances (decompensated heart failure, shock, acute myocardial ischemia, etc.), living in nursing homes or palliative care units, and refusal to participate in the study by patients or their legal representatives. Additionally, patients living in nursing homes or receiving palliative care were not included in the study due to legal restrictions. Due to the nature of the neuropsychiatric test evaluation, only samples with formal education were included in the analysis. Furthermore, patients who were bedridden or in the latest stage of dementia were not admitted to the outpatient clinic by their caregivers and therefore had to be excluded from the study.

### 2.2. Statistical analysis

In this study, categorical data were summarized as count with percent, while continuous data were summarized by using mean ± standard deviation (if normality was assumed) and median (min-max) values. The one-way ANOVA was employed to compare mean ages and mean symptom durations of four groups. The chi-square statistics were used to analyze the categorical demographic and clinical features of groups. Repeated measurements ANOVA and Friedman ANOVA were utilized to evaluate the neuropsychiatric change of the cases with AD from admittance to the last visit. The association between gender and some clinical characteristics of AD such as ApoE, hippocampal atrophy, Fazekas grading, epilepsy, and Parkinsonism were also evaluated using chi-square or likelihood ratio test (for small expected frequencies) statistics.

This study aimed to explore the differentiation in the prognosis of patients with AD and the risk factors affecting this differentiation. To achieve this aim, we used group-based trajectory models, an approach used for identifying distinctive patient clusters and profiling the characteristics of individuals within the clusters. The outcomes of the model were GDS-I, GDS-II, and GDS-III repeatedly measured at three times (admittance, second visit, last visit), and risk factors (or covariates) were Fazekas, hippocampal, BDLAS, EDLAS, comorbid medical conditions, and family history of dementia. The reliability of the model was evaluated using entropy-based goodness of fit statistics, and the model was assumed reliable if the entropy value was greater than 0.80.

The group-based trajectory modeling analysis was conducted using the STATA Plugin (29), while the other analyses were conducted using STATISTICA 13.0 (30).

## 3. Results

**Figure 1:**
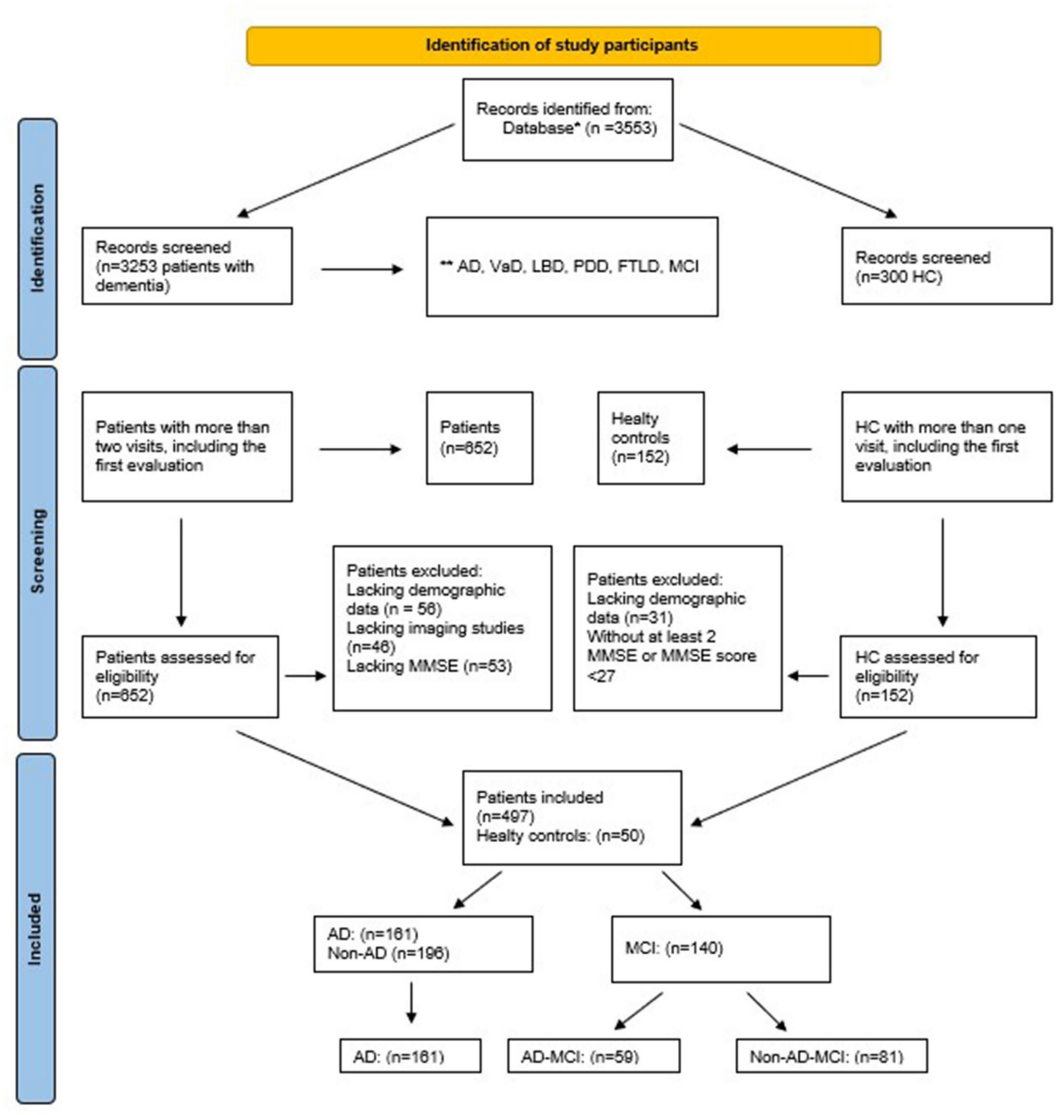
Flowchart of study sample.*Epikriz.com (Turkish Alzheimer Database, Mersin Branch). **AD, Alzheimer’s disease, VaD, vascular dementia, LBD, lewy body dementia, PDD, Parkinson’s disease with dementia, FTLD, frontotemporal dementia, MCI, mild cognitive impairment, MMSE, mini-mental state examination, HC, healthy controls.

This study analyzed 3,553 cases from the database, and after undergoing an extensive evaluation process, 161 clinical cases with AD were included for prognostic analysis. More details about the selection process can be seen in Figure 1.

The mean age of the study population was 73.16 ± 7.23 years, with a female predominance (68.32%). Our AD population was slightly older than the non-AD population (<0.001). Gender distribution differed among the groups, with the prodromal dementia group having a higher proportion of females (55.71%) compared to males (44.28%). In contrast, the Alzheimer’s disease group had a higher proportion of males (68.32%) compared to females (31.68%) (*p* = 0.00784).

After the first visit, we followed all cases throughout their lifetime. It should be noted that this analysis only included cases with sufficient neuropsychiatric evaluation and other complementary data. As shown in Table 1, the mean duration of follow-up evaluation after the first visit was 12.50 ± 11.64 months, and the last visit mentioned in the analysis was 25.71 ± 18.68 months. Interestingly, the interval between the second and third visit was longer than the first and second visits (*p* < 0.001). Although we have some cases with longer follow-up periods, the maximum follow-up duration of the analysis was 211 months, and we could not include many cases referred to nursing homes or bedridden individuals who were unable to attend regular outpatient visits.

**Table 1:** Demographic and clinical features of study sample

	Healthy control *n*=50 (9.14%)	Prodromal dementia (MCI) *n*=140 (25.59%)	Alzheimer’s disease *n*=161 (29.44%)	Non-Alzheimer dementias *n*=196 (35.83%)	Total *n*=547	*p*
Age. year (mean ±SD)	65.59 ±9.96	70.44 ± 6.94	73.16 ± 7.23	71.02 ± 9.13		<0.001
Follow up duration (months. mean±SD)						
First visit Second visit Last visit	28.32 ± 35.11 (0-120) – 26.72 ± 27.58 (4-127)	27.96 ± 26.68 (3-120) 14.17 ± 12.98 (3-79) 35.45 ± 34.51 (12-203)	33.44 ± 28.07 (3-120) 12.50 ± 11.64 (1-68) 25.71 ± 18.68 (2-93)	28.93 ± 25.50 (3-120) 10.72 ± 9.90 (3-90) 22.92 ± 17.91 (3-94)	29.96 ± 27.57 (0-120) 12.27 ± 11.46 (1-90) 27.29 ± 24.76 (2-174)	
Gender						
Female *n* (%) Male *n* (%)	28 (56%) 22 (44%)	78 (55.71%) 62 (44.28%)	110 (68.32%) 51 (31.68%)	99 (50.51%) 97 (49.49%)	315 (57.59%) 232 (42.41%)	0.00784
Formal education, *n* (%)						
Basic school Middle school High school University	42 (84%) – – 8 (16%)	120 (85.71%) 4 (2.86%) 5 (3.57%) 11 (7.86%)	142 (88.20%) 8 (4.97%) 3 (1.86%) 8 (4.97%)	183 (93.37%) 3 (1.53%) 2 (1.02%) 8 (4.08%)		0.01886
Living						
Alone with family	3 (6%) 47 (94%)	23 (16.43%) 117 (83.57%)	33 (20.50%) 128 (79.50%)	33 (16.84%) 163 (83.16%)	92 (16.82%) 455 (83.18%)	0.12414
Presentation symptoms *n* (%)						
Memory dysfunction Language dysfunction Executive dysfunction Behavioral problem Self-care problem Sleep problem Disorientation Incontinence Loss of appetite		51 (36.43%) 85 (60.71%) 19 (13.57%) 2 (1.43%) 7 (5%) 41 (29.28%) 33 (23.57%) 28 (20%) 16 (11.43%)	82 (50.93%) 117 (72.67%) 64 (39.75%) 16 (9.94%) 35 (21.74%) 67 (41.61%) 82 (50.93%) 44 (27.33%) 40 (24.84%)	82 (41.84%) 133 (67.86%) 61 (31.12%) 7 (3.57%) 48 (24.49%) 76 (38.77%) 81 (41.33%) 48 (24.49%) 35 (17.86%)		0.01848 0.00295 <0.00001 0.00084 <0.00001 0.13841 <0.00001 0.05862 0.00919
Comorbid medical problems *n* (%)						
Hypertension Thyroid dysfunc. Diabetes mellitus CAD Hyperlipidemia Stroke Current smoker No smoker Quit smoker Regular alcoholic Non-alcoholic Quit alcoholic	19 (38%) 7 (14%) 9 (18%) 12 (24%) 9 (18%) 1 (2%) 7 (14%) 32 (64%) 11 (22%) – 47 (94%) 3 (6%)	60 (42.86%) 22 (15.71%) 30 (21.43%) 38 (27.14%) 34 (24.28%) 6 (4.28%) 18 (12.86%) 88 (62.86%) 34 (24.28%) 15 (10.72%) 122 (87.14%) 3 (2.14%)	79 (49.07%) 14 (8.69%) 36 (22.36%) 35 (21.74%) 35 (21.74%) 6 (3.73%) 8 (4.97%) 119 (73.91%) 34 (21.12%) 13 (8.08%) 144 (89.44%) 4 (2.48%)	89 (45.41%) 23 (11.73%) 38 (19.39%) 56 (28.57%) 39 (19.90%) 12 (6.12%) 15 (7.65%) 138 (70.41%) 43 (21.94%) 14 (7.14%) 172 (87.75%) 10 (5.10%)	247 (45.15%) 66 (12.06%) 113 (24.04%) 141 (20.66%) 117 (21.39%) 25 (4.57%) 48 (8.77%) 377 (68.93%) 122 (22.30%) 42 (7.68%) 485 (88.66%) 20 (3.66%)	0.50628 0.29858 0.86285 0.49531 0.72651 0.54613 0.16899 – – 0.24199 – –
Epilepsy	–	18 (12.86%)	22 (13.66%)	49 (25.0%)	89 (16.27%)	0.00005
Extrapyramidal symptoms	–	19 (13.57%)	18 (11.18%)	64 (32.65%)	102 (18.65%)	<0.00001
Family history of dementia *n* (%) Family history of vascular disease	18 (36%) 23 (46%)	61 (43.57%) 73 (52.14%)	59 (36.64%) 56 (34.78%)	82 (41.84%) 76 (38.77%)	220 (40.22%) 228 (41.68%)	0.55337 0.01463

*, Statistically significant according to Bonferroni adjusted *p* value (0.00625).

**, First visit duration means that duration of the first presenting symptom.

Our study sample showed a significant female predominance in each diagnostic subgroup (*p* < 0.007). Most participants had completed at least basic or middle school education, and there was a significant difference in formal education levels between the groups (*p* = 0.012). Due to the exclusion of patients living in nursing homes and barriers to regular visits to the government hospital, many subjects reported living with family.

The most common presentation symptom in all three groups was memory dysfunction, followed by language dysfunction and executive dysfunction. Interestingly, behavioral problems were more common in participants with Alzheimer’s disease compared to the other groups (*p* = 0.001). Among the presentation symptoms, self-care problem and sleep problem were relatively less common compared to the other symptoms in all three groups. Disorientation and incontinence were more prevalent in participants with non-Alzheimer dementias, while loss of appetite was more prevalent in participants with Alzheimer’s disease. These findings suggest that different types of dementia may have distinct presentation symptoms and that these symptoms may have different underlying mechanisms.

There were no significant differences in the prevalence of comorbid medical problems among the groups. However, the prevalence of both epilepsy and extrapyramidal symptoms was highest in the non-Alzheimer dementias group (25.0 and 32.65%, respectively, *p* < 0.001).

Regarding family history, there was no significant difference between the groups in terms of family history of dementia, but there was a significant difference for vascular diseases (*p* < 0.001).

The analysis shows that the duration between the second and third visits was longer than the duration between the first and second visits. In details, Table 2 showed significant functional deterioration measured by BDLAS and EDLAS (*p* < 0.001). The mean MMSE scores were 21.30 ± 6.43 at the acceptance, and they significantly progressed (20.63 ± 7.74 and 15.99 ± 8.17, *p* < 0.001). The mean digit forward ratio was 4 points for each visit, and backward was 2 points, but they worsened significantly (*p* = 0.015 for digit forward and *p* = 0.008 for backward). Calculation was tested in educated AD cases, and the scores also decreased significantly along the process (*p* < 0.001). Abstraction was also widely tested and showed regression in the follow-up visits (*p* < 0.001). WMT step 1–2-3 and recognition deteriorated significantly along the process (*p* = 0.058, *p* = 0.038, *p* = 0.012, and *p* = 0.006, respectively). BNT and comprehension were other significant predictors of the prognosis (*p* < 0.001). CDT also decreased step by step significantly (*p* = 0.017). Visual memory score and recall could only be performed on educated cases, and the scores also decreased along with the follow-up visits (*p* = 0.042 and *p* = 0.013, respectively). As a specific global deterioration presenter, the average GDS was 3 at the admittance. It progressed to 4 at the second visit and then significantly progressed to 5 on the last visit included in this study protocol (*p* < 0.001). Overall, these results suggest a decline in cognitive and memory abilities as dementia progresses, which may be useful in monitoring disease progression.

**Table 2:** Neuropsychiatric evaluation of the cases with AD.

	First visit	Second visit	Last visit	*p*
BDLAS	2.76 ± 1.97 *n*=149 (0-8)	2.89 ± 2.23 *n*=114 (0-8)	4.12 ± 2.22 *n*=97 (0-8)	<0.001
EDLAS	15.19 ± 6.85 *n*=144 (0-23)	14.48 ± 7.98 *n*=113 (0-23)	9.69 ± 7.74 *n*=96 (0-23)	<0.001
MMSE	21.30 ± 6.43 *n*=161 (0-30)	20.63 ± 7.74 *n*=161 (0-30)	15.99 ± 8.17 *n*=161(0-30)	<0.001
Digit forward Digit backward	4 (0-6) *n*=136 2 (0-5) *n*=132	4 (0-10) *n*=126 2 (0-5) *n*=121	4 (0-7) *n*=121 2 (0-5) *n*=116	0.015 0.008
Calculation	5 (0-5) *n*=128	5 (0-5) *n*=113	2 (0-5) *n*=111	<0.001
Abstraction	3 (0-3) *n*=134	3 (0-3) *n*=119	3 (0-3) *n*=121	<0.001
WMT- step 1 (max:10) WMT-step 2 (max:10) WMT-step 3 WMT- recall (max:10) WMT-recognition (max:20)	2 (0-6) *n*=124 3 (0-8) *n*=128 3 (0-8) *n*=127 0 (0-7) *n*=121 14 (0-20) *n*=121	2 (0-7) *n*=116 3 (0-7) *n*=115 3 (0-9) *n*=116 0 (0-8) *n*=111 13 (0-20) *n*=115	1 (0-6) *n*=114 2 (0-9) *n*=113 2 (0-10) *n*=112 0 (0-10) *n*=50 10 (0-20) *n*=112	0.058 0.038 0.012 0.347 0.006
BNT	11 (0-15) *n*=130	12 (0-15) *n*=117	10 (0-15) *n*=115	<0.001
Comprehension	6 (0-6) *n*=95	6 (0-6) *n*=73	3 (0-6) *n*=83	<0.001
CDT	4 (0-10) *n*=126	5 (0-10) *n*=110	3 (0-10) *n*=113	0.017
Visual memory score	5 (0-11) 50	4 (0-11) *n*=47	5 (0-11) *n*=55	0.042
Visual memory recall	1 (0-11) *n*=48	0 (0-11) *n*=45	0 (0-11) *n*=50	0.013
GDS	3 (3-6) *n*=161	4 (3-7) *n*=161	5 (3-7) *n*=161	<0.001

Descriptive values are mean ±SD or median (min-max).

BDLAS, Barthel Daily Living Activities Scale; EDLAS, Elderly Daily Living Activity Scale; MMSE, Minimental State Examination; WMT, Word memory test; BNT, Boston naming test; CDT, Clock Driving Test; GDS, Global Deterioration Scale.

*, Statistically significant according to Bonferroni adjusted *p* value (0.0027).

**, First visit duration means that duration of the first presenting symptom.

Due to technical issues provided by our institute, there is limited data about ApoE genotype, but there is comprehensive information about hippocampal atrophy and WMIs graded by Fazekas, as shown in Table 3. There is no gender difference in the mentioned parameters. Most of our cases had Grade 2 (50.31%) or Grade 3 (32.30%) level of hippocampal atrophy at admission. On the other hand, only 28.57% of our AD cases had no mentioned WMIs (Fazekas 0), but more than half (54.66%) scored Grade 1, and the remaining (16.77%) had WMIs scored as Fazekas 2. None of the cases were evaluated as Fazekas 3 in the study sample.

**Table 3:** Laboratory evaluation of the cases with AD.

	Female (Total=110)	Male (Total=51)	Total (Total=161)	*p*
APOE genotype *n* (%)				
E3/E4 E2/E4 E3/E3 E4/E4	8(61.54%) 1(7.69%) 3(23.08%) 1(7.69%)	5(50%) – 3(30%) 2(20%)	13(56.52%) 1(4.35%) 6(26.09%) 3(13.04%)	0.56574
Hippocampal atrophy *n* (%)				
Grade 1 Grade 2 Grade 3	22(20.00%) 54(49.09%) 34(30.91%)	6(11.76%) 27(52.94%) 18(35.29%)	28(17.39%) 81(50.31%) 52(32.30%)	0.43410
Fazekas scores *n* (%)				
Grade 0 Grade 1 Grade 2	33(30.00%) 60(54.55%) 17(15.45%)	13(25.49%) 28(54.90%) 10(19.61%)	46(28.57%) 88(54.66%) 27(16.77%)	0.73813

**Figure 2:**
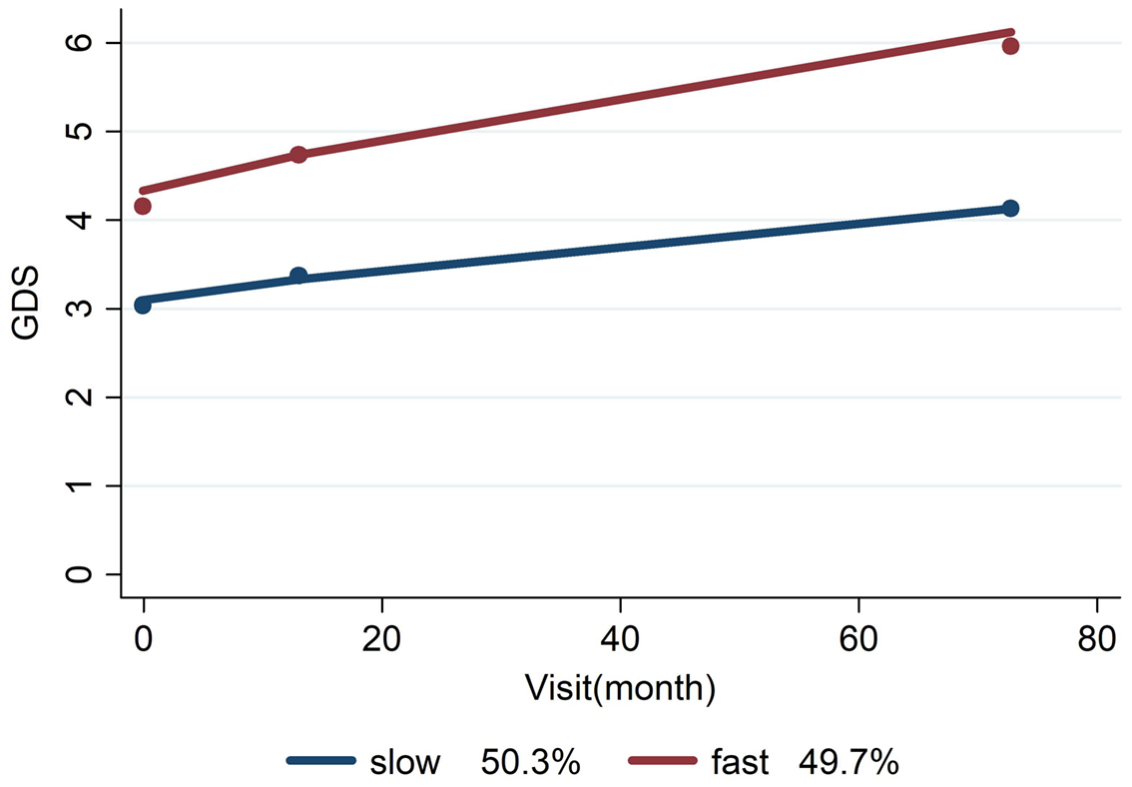
Slow/fast prognosis of AD.

In order to model and determine potential stakeholders in disease progression, we performed group-based trajectory analysis. This analysis showed that AD cases separated into two reliable subgroups as Slow/Fast prognosis with high reliability (50.3% vs. 49.7%, Entropy = 0.837), as shown in Figure 2.

The cases with mean GDS score level 3 or below at admission had slow progression, but those with level 4 or higher had a fast prognosis. Comprehensive cofactors of the mentioned progression are listed in Table 4, and as shown in the table, one of the most important predictors of progression is hippocampal atrophy (Coeff. = 1.18775, *p* = 0.0001). High levels of hippocampal atrophy were closely related to a high progression rate in our study sample with AD. As shown in Table 4, around 48.78% of the AD cases with fast progression had level 3 or higher hippocampal atrophy. There is also a significant association between AD progression and Fazekas grading (Coeff. = 0.91490, *p* = 0.0029). On the other hand, functional disabilities scored with BDLAS/EDLAS (Coeff. = 0.61847, *p* < 0.0001/ Coeff. = −0.20638, *p* < 0.0001, respectively) are other important predictors of AD progression. A high level of functional deterioration was closely related to fast AD progression.

**Table 4:** Trajectory model coefficients, standard errors, and significances of risk factors for AD prognosis with descriptive statistics [*n* (%), mean (SD), (min-max)].

	Slow progression *n*=81 (50.3%)	Fast progression *n* 80 (49.7%)	Risk		
Fazekas grading			Coeff.	Std. Error	*p*
0	30 (37.97%)	16 (19.51%)	0.91490	0.30555	0.0029
1	43 (54.43%)	45 (54.88%)			
2	6 (7.59%)	21 (25.61%)			
Hippocampal atrophy					
1	20 (25.32%)	8 (9.76%)	1.18775	0.29834	0.0001
2	47 (59.49%)	34 (41.46%)			
3	12 (15.19%)	40 (48.78%)			
BDLAS First visit	1.936±1.307 (0-7.5)	3.482±2.174 (0-8)	0.61847	0.13864	<0.001
BDLAS Second visit	1.706±1.301 (0-7)	3.841±2.365 (0-8)			
BDLAS Last Visit	2.780±1.743 (0-7.5)	5.107±2.011 (1-8)			
EDLAS First visit	18.662±4.943 (0-23)	12.079±6.852 (1-23)	–0.20638	0.04029	<0.001
EDLAS Second visit	18.72±5.167 (0-23)	11.11±8.29 (0-23)			
EDLAS Last visit	14.45±7.749 (0-23)	6.286±5.713 (0-23)			
Epilepsy					
No	71 (89.87%)	68 (82.93%)	0.65964	0.56549	0.2440
Yes	8 (10.13%)	14 (17.07%)			
Parkinsonism					
No	73 (92.41%)	70 (85.37%)	0.76813	0.62767	0.2216
Yes	6 (7.59%)	12 (14.63%)			
Thyroid dysfunction					
No	70 (88.61%)	77 (93.90%)	–0.22062	0.66721	0.7411
Yes	9 (11.39%)	5 (6.10%)			
CAD					
No	59 (74.68%)	67 (81.71%)	–0.27174	0.49035	0.5797
Yes	20 (25.32%)	15 (18.29%)			
DM					
No	61 (77.22%)	64 (78.05%)	0.21251	0.47760	0.6566
Yes	18 (22.78%)	18 (21.95%)			
Hypertension					
No	39 (49.37%)	43 (52.44%)	0.17567	0.40802	0.6670
Yes	40 (50.63%)	39 (47.56%)			
History of stroke					
No	76 (96.20%)	79 (96.34%)	0.11309	0.94267	0.9046
Yes	3 (3.80%)	3 (3.66%)			
Hyperlipidemia					
No	58 (73.42%)	68 (82.93%)	–0.70698	0.49441	0.1534
Yes	21 (26.58%)	14 (17.07%)			
Smoking					
Current	3 (3.80%)	5 (6.10%)	–0.09225	0.36071	0.7983
No	60 (70.95%)	59 (71.95%)			
Ex smoker	16 (20.25%)	18 (21.95%)			
Regular alcohol use					
Current	6 (7.59%)	7 (8.54%)	–0.47912	0.55863	0.3915
No	69 (87.34%)	75 (91.46%)			
Ex user	4 (5.06%)	0 (0%)			
Family history of dementia					
No	48 (60.76%)	54 (65.85%)	–0.27642	0.35897	0.4416
Yes	31 (39.24%)	28 (34.15%)			

Additionally, we performed a cut-off analysis for BDLAS/EDLAS with ROC analysis. Our data showed that cases with >3 (Sensitivity = 79.8%, Specificity = 51.4%) functional disability detected by BDLAS had fast progression, but those with >14 scores of EDLAS (Sensitivity = 59.2%, Specificity = 83.8%) had a slow prognosis. Moreover, the predictive role of BDLAS was performed after admission, but EDLAS showed an effect after the second visit as a predictive stakeholder.

These results indicate that Fazekas grading and hippocampal atrophy are significant risk factors for Alzheimer’s disease progression (*p* = 0.0029 and *p* = 0.0001, respectively). BDLAS at the first visit and EDLAS at all visits were also found to be significant predictors of disease progression (*p* < 0.001). Other comorbidities such as Parkinsonism, thyroid dysfunction, CAD, DM, hypertension, history of stroke, hyperlipidemia, smoking, regular alcohol use, and family history of dementia did not show a significant association with Alzheimer’s disease progression in this analysis. However, it should be noted that the sample size for some of these categories may be small, and further research with larger sample sizes may be needed to confirm these findings.

## 4. Discussion

The present study aimed to evaluate potential predictors of Alzheimer’s disease (AD) progression using a comprehensive follow-up dataset, including functional/cognitive assessments, clinical and neuropsychiatric evaluations, and neuroimaging biomarkers such as hippocampal atrophy or white matter intensities (WMIs). The study group consisted of 161 AD cases recruited from 3,553 cases in a dementia database, consisting of individuals who had consulted the Dementia Outpatient Clinic of the Neurology Department at Mersin University Medical Faculty between 2000 and 2022, under the supervision of the same senior author (AO). The results showed that there were two reliable subgroups of AD cases with slow and fast progression, with high sensitivity (Entropy = 0.836). The study also identified several important predictors of AD progression, such as hippocampal atrophy, functional disabilities, and Fazekas grading. These findings have significant implications for early detection, diagnosis, and treatment of AD, as well as for developing strategies to improve the quality of life for individuals with this disease.

Research has shown that the progression of Alzheimer’s disease may differ based on gender. Studies suggest that women may experience a faster rate of disease progression than men (31). One study found that women with Alzheimer’s disease have a higher rate of cognitive decline than men, even after controlling for factors such as age, education level, and baseline cognitive function (32). Another comprehensive meta-analysis reported that women with Alzheimer’s disease have greater brain atrophy than men, which is a hallmark of the disease (33). Our study revealed that there was a female predominance of 68.32%, but gender did not have any significant effect on the prognosis as a predictor.

Research has demonstrated that the symptoms of Alzheimer’s disease can vary depending on the age and stage of the disease. Younger individuals with Alzheimer’s disease may be more prone to changes in behavior and personality, while older individuals may experience more severe cognitive decline (34). Memory loss is a key symptom of Alzheimer’s disease, with individuals having difficulty remembering recent events or conversations, forgetting names of familiar people or objects, or repeating themselves often. As the disease progresses, memory loss can worsen and eventually impair the individual’s ability to recognize even close family members. In our study, we found that only 66.13% of our sample had presented with memory problems, while 78.52% had language problems such as naming difficulties. Executive dysfunction is also known to be affected by AD and can impact an individual’s ability to plan, organize, and execute tasks, although it is not a common presenting symptom. Individuals may have difficulty making decisions or solving problems, and may become easily overwhelmed by complex tasks, as seen in 43.24% of our AD cases. Additionally, we found that only 11.03% of our subjects presented with behavioral changes such as agitation, aggression, and disinhibition, which can be a shadow presenting symptom. As the disease progresses, individuals with Alzheimer’s disease may have difficulty performing activities of daily living, such as bathing, dressing, and grooming, as seen in 23.65% of our cases. Sleep disturbances are common in AD, with individuals experiencing disrupted sleep–wake cycles, increased daytime sleepiness, and frequent waking during the night, which was present in 45.27% of our patients. More than half of our cases (55.41%) presented with difficulty perceiving and navigating the physical environment. Individuals may have difficulty with depth perception, object recognition, and spatial orientation, and may become disoriented as the disease progresses, losing track of time, place, and person. Loss of bladder and bowel control can also occur as the disease progresses, particularly in the later stages, as seen in 29.93% of our cases in the admittance. Individuals with Alzheimer’s disease may also experience a loss of appetite, leading to weight loss and other health complications, which was present in 27.03% of our sample (31, 35, 36).

Some studies have suggested that smoking may influence disease progression in individuals with Alzheimer’s disease. Smokers with AD may experience more rapid cognitive decline than non-smokers, with an average decline of 2.7 points per year on the MMSE, compared to 1.8 points per year in non-smokers. However, it is important to note that the impact of smoking on disease progression is still controversial and further research is needed to fully understand the relationship between smoking and Alzheimer’s disease (37). Our study showed that there was no significant association between smoking and AD progression. Similarly, there is a complex and not yet fully understood relationship between alcohol use and Alzheimer’s disease progression, which is not supported by our data (38). Overall, while some research claims that moderate alcohol consumption may have protective effects against cognitive decline, heavy alcohol use is associated with an increased risk of Alzheimer’s disease and other forms of dementia, which requires further comprehensive analysis.

It is known that medical comorbidities are common in individuals with Alzheimer’s disease and can impact disease progression and outcomes. Studies have shown that individuals with Alzheimer’s disease who have medical comorbidities tend to experience more rapid cognitive decline, functional impairment, and higher mortality rates than those without comorbidities (39). One of the most common medical comorbidities in individuals with Alzheimer’s disease is cardiovascular disease. Research has shown that cardiovascular risk factors, such as hypertension, diabetes, and hyperlipidemia, are associated with an increased risk of developing Alzheimer’s disease and may also accelerate disease progression (40). As unusual information, our study revealed that a high frequency of medical comorbidities, particularly vascular and metabolic problems, are common in the AD population requires a comprehensive explanation but no effect on the progression.

A positive family history of dementia, particularly in first-degree relatives, is a well-established risk factor for both the development and rapid cognitive decline of Alzheimer’s disease progression (41). However, our sample (39.24% vs. 34.15%, *p* = 0.4416) did not support this association.

While ongoing research investigates the use of neuropsychiatric tests as prognostic biomarkers for Alzheimer’s disease, there is currently no consensus on which tests are the best predictors of disease progression. Several studies have investigated the use of neuropsychiatric tests to predict Alzheimer’s disease progression. For example, one study found that measures of verbal memory and executive function were predictive of future cognitive decline in individuals with mild cognitive impairment (42). Another study reported that measures of visuospatial abilities and attention were predictive of cognitive decline in individuals with early-stage Alzheimer’s disease (43). Our study demonstrated that functional disability tests have a predictive role in differentiating the AD progression. Among the frequently used screening tests, MMSE, digit span test, calculation, comprehension, BNT, CDT, and visual memory tests showed significant progression during the follow-up process. However, the WMT-first step, which is an important predictor not only for the diagnosis but also for the progression of AD, did not show any significant difference along the process. In contrast, the results of step 2 and step 3 tests showed significant differences in the prognosis. It is possible that due to the preserved neural network, WMT-recall did not show any difference, but recognition was an important predictor of the progression, as indicated in Table 2. In the aspect of cumulative evaluation of the neuropsychiatric batteries we can say that scores decreased significantly as the disease progressed, as evidenced by a significant decrease in MMSE, Digit Forward, Digit Backward, Calculation, and Abstraction scores. The scores for the WMT showed a significant decrease in Step 3 and recall, while recognition scores did not change significantly. The Boston Naming Test (BNT) and Comprehension test scores also decreased significantly as the disease progressed. The Clock Drawing Test (CDT) scores decreased significantly from the first visit to the last visit. Visual Memory Score also showed a significant decrease from the first to the last visit, while the Visual Memory Recall score decreased significantly between the first and second visit. Finally, GDS scores increased significantly from the first visit to the last visit. The battery used in the study is designed to assess various cognitive functions, including memory, language, executive functions, and daily living activities, but it may not have specific tests to thoroughly evaluate judgment and visuospatial function. It is crucial to highlight the potential for future research to further enhance our understanding of Alzheimer’s disease progression by incorporating specialized tests that specifically target judgment and visuospatial function. Our data also indicates the prognostic importance of functional capacity evaluation as an under-evaluated parameter.

Hippocampal atrophy, characterized by a loss of volume in the hippocampus region of the brain, is a well-established biomarker for Alzheimer’s disease but the importance on the prognosis is challenging issue. One study found that individuals with mild cognitive impairment who had greater hippocampal atrophy were more likely to progress to Alzheimer’s disease than those with less atrophy (44). Another study reported that hippocampal atrophy was associated with more rapid cognitive decline in individuals with Alzheimer’s disease (45). In addition to predicting disease progression, hippocampal atrophy may also have value as a diagnostic biomarker for Alzheimer’s disease. One study reported that the combination of clinical evaluation and hippocampal volume measurements resulted in a higher diagnostic accuracy for Alzheimer’s disease than either measure alone (46). Our study showed that most of the AD patients had Grade 2 (50.31%) or Grade 3 (32.30%) level of hippocampal atrophy at admission. When evaluated all the predictors altogether, the most important predictor of progression is hippocampal atrophy (Coeff. = 1.31975, *p* < 0.001). High levels of hippocampal atrophy were closely related to a high progression rate in our study sample with AD. As shown in Table 4, around 53.33% of the AD cases with fast progression had level 3 or higher hippocampal atrophy. Overall, hippocampal atrophy is a well-established biomarker for Alzheimer’s disease, with significant diagnostic and prognostic value.

Fazekas grading is a neuroimaging technique used to measure white matter hyperintensities (WMH) in the brain, which are associated with several neurological disorders including Alzheimer’s disease. WMH are areas of increased signal intensity on T2-weighted magnetic resonance imaging (MRI) scans, and can be graded using the Fazekas scale, which ranges from 0 to 3 based on the severity and extent of WMH (14). Several studies have investigated the use of Fazekas grading as a biomarker for Alzheimer’s disease. One study reported that combining Fazekas grading with other biomarkers, such as beta-amyloid and tau proteins in cerebrospinal fluid, resulted in a higher diagnostic accuracy for Alzheimer’s disease than using Fazekas grading alone (47). However, there is currently no longitudinal data available on the prognostic value of white matter hyperintensities (WMIs) in AD progression. Our study found that only 28.57% of AD cases had no mentioned WMIs (Fazekas 0), while more than half (54.66%) had Grade 1 WMIs and the remaining (16.77%) had WMIs scored as Fazekas 2. Additionally, we found a significant association between AD progression and Fazekas grading (Coeff. = 0.74012, *p* = 0.0164). However, Fazekas grading does not have a clear accumulation like hippocampal atrophy in our study sample. Inclusive, Fazekas grading is a well-established neuroimaging technique that can provide valuable information about WMIs in the brain and may be a promising prognostic factor.

Apolipoprotein E (ApoE) genotype has been extensively studied in the context of Alzheimer’s disease as a potential genetic risk factor. Multiple studies have investigated the relationship between ApoE genotype and Alzheimer’s disease progression. One study found that individuals with mild cognitive impairment who were carriers of the ε4 allele had a higher risk of progressing to Alzheimer’s disease than non-carriers (48). Another study reported that ε4 carriers had a faster rate of cognitive decline and greater hippocampal atrophy compared to non-carriers in the early stages of Alzheimer’s disease (49). Due to the insurance system not covering the ApoE genotype studies, we have limited data in our dataset. Our limited data supported that there is no significant effect of ApoE genotype on gender or disease progression, neither alone nor as a stakeholder.

Recent research has suggested that Alzheimer’s disease (AD) may have two distinct subtypes, with different rates of disease progression and underlying biological mechanisms. These subtypes have been labeled as fast and slow progressive AD. Fast progressive AD is characterized by a more rapid rate of cognitive decline, with individuals experiencing significant cognitive impairment within a few years of disease onset. One study found that individuals with fast progressive AD had higher levels of neuroinflammation and a greater burden of amyloid and tau pathology compared to those with slow progressive AD (50). Slow progressive AD, on the other hand, is characterized by a slower rate of cognitive decline, with individuals maintaining a higher level of cognitive function for a longer period. This subtype has been associated with greater resilience and plasticity of brain networks, allowing individuals to better compensate for the effects of pathological changes in the brain (51). The identification of fast and slow progressive subtypes of Alzheimer’s disease (AD) may have significant implications for clinical management and treatment. Individuals with fast progressive AD may require more aggressive interventions to slow disease progression, such as early initiation of disease-modifying therapies or targeted treatments to reduce neuroinflammation. On the other hand, individuals with slow progressive AD may benefit more from interventions focused on maintaining cognitive function and promoting neuroplasticity. Our data revealed that the AD cases were separated into two reliable subgroups as Slow/Fast prognosis with high sensitivity (Entropy = 0.836), as shown in Figure 2. We also demonstrated the significant effect of hippocampal atrophy (Coeff. = 1.31975, *p* < 0.001), WMIs with Fazekas grading (Coeff. = 0.74012, *p* = 0.0164), functional disabilities scored with BDLAS/EDLAS (Coeff. = 0.75249, p < 0.001/Coeff. = −0.25223, p < 0.001, respectively), which had a borderline predictor of fast AD progression (Coeff. = 1.26267, *p* = 0.0522). While the concept of fast and slow progressive AD is still in its early stages, it has the potential to improve our understanding of the underlying mechanisms of AD and inform personalized treatment approaches. However, further research is needed to validate these subtypes and identify reliable biomarkers for their early identification.

### 4.1. Implications

Based on the findings of the study, there are several implications that can be drawn:

The identification of fast and slow progressive subtypes of AD has important implications for clinical management and treatment. Early detection and intervention for fast progressive AD may be crucial in slowing the disease progression and improving patient outcomes.

The presence of medical comorbidities, particularly cardiovascular disease, may have a significant impact on AD progression and management. Therefore, the management of comorbidities should be considered in the treatment plan for patients with AD.

The study found no significant association between smoking and AD progression, and the relationship between alcohol use and AD progression remains complex and not fully understood.

Neuropsychiatric tests, particularly measures of verbal memory and executive function, can be useful in predicting future cognitive decline in individuals with AD.

The BDLAS and EDLAS are commonly used tools to assess functional disabilities in elderly individuals. In the context of Alzheimer’s disease, these tools can provide valuable information on the prognosis of the disease.

The presence of white matter hyperintensities, as measured by Fazekas grading, similar to hippocampal atrophy may be a promising prognostic factor for AD progression.

Finally, the findings of the study contribute to our understanding of the factors that impact AD progression and can inform personalized treatment approaches for patients with AD.

### 4.2. Limitations of the study

The study only included individuals who had consulted the Dementia Outpatient Clinic of the Neurology Department at Mersin University Medical Faculty, which may not be representative of the general population.

The study only focused on predictors of AD progression, and did not examine potential interventions or treatments for the disease.

The study did not examine the impact of other demographic factors, such as race/ethnicity or socioeconomic status, dietary habituation, social support, etc. on AD progression.

The study relied on self-reported or caregiver-reported symptoms, which may be subject to recall bias or other reporting biases.

The study did not examine the impact of genetic factors on AD progression, which is known to play a role in the disease.

The study only evaluated the predictive role of functional/cognitive assessments and did not include other potential predictors of Alzheimer’s disease progression, such as genetic markers or lifestyle.

The study did not examine the impact of smoking or alcohol use on disease progression over time, as it was only analyzed at the initial assessment.

The sample size of the study may not be representative of the broader population of individuals with Alzheimer’s disease, and further research with larger sample sizes is needed to validate the identified subtypes.

The study did not investigate the impact of the identified subtypes on other outcomes, such as mortality, quality of life, or caregiver burden, which could be relevant for clinical management and treatment decisions.

The study did not examine the impact of different treatment approaches on the identified subtypes, and further research is needed to investigate the effectiveness of personalized treatment approaches based on the fast and slow progression subtypes of Alzheimer’s disease.

## 5. Conclusion

In conclusion, the present study evaluated potential predictors of AD progression using a comprehensive follow-up dataset, including functional/cognitive assessments, clinical and neuropsychiatric evaluations, and neuroimaging biomarkers such as hippocampal atrophy or white matter intensities (WMIs). The study identified two reliable subgroups of AD cases with slow and fast progression, with high sensitivity (Entropy = 0.836), and several important predictors of AD progression, such as hippocampal atrophy, functional disabilities, and Fazekas grading. This study demonstrated that among medical comorbidities that create vascular risk, particularly smoking and regular alcohol use are significant risk factors that can be monitored and supported with the Fazekas scale, and they have an impact on the prognosis. The findings have significant implications for early detection, diagnosis, and treatment of AD, as well as for developing strategies to improve the quality of life for individuals with this disease. The identification of fast and slow progressive subtypes of Alzheimer’s disease may also inform personalized treatment approaches. However, further research is needed to validate these subtypes and identify reliable biomarkers for their early identification.

## Data availability statement

The data analyzed in this study is subject to the following licenses/restrictions: this is only accessible to doctors who are members of the system. Requests to access these datasets should be directed to http://www.epikriz.com/index.aspx.

## Ethics statement

Ethical review and approval was not required for the study of human participants in accordance with the local legislation and institutional requirements. Written informed consent from participants was not required to participate in this study in accordance with the national legislation and the institutional requirements.

## Author contributions

AÖ: data collection, conception, design, revision of the article, and approved the final version. RG and NÖ: data collection. AÖ, RG, NÖ, and BT: analysis and drafting of the article. All authors contributed to the article and approved the submitted version.

## Conflict of interest

The authors declare that the research was conducted in the absence of any commercial or financial relationships that could be construed as a potential conflict of interest.

## Publisher’s note

All claims expressed in this article are solely those of the authors and do not necessarily represent those of their affiliated organizations, or those of the publisher, the editors and the reviewers. Any product that may be evaluated in this article, or claim that may be made by its manufacturer, is not guaranteed or endorsed by the publisher.

## Footnotes

^1^www.epikriz.com/dementiadataset
